# Efficacy of Superselective Conventional Transarterial Chemoembolization Using Guidance Software for Hepatocellular Carcinoma within Three Lesions Smaller Than 3 cm

**DOI:** 10.3390/cancers13246370

**Published:** 2021-12-19

**Authors:** Shiro Miyayama, Masashi Yamashiro, Rie Ikeda, Junichi Matsumoto, Kiyotaka Takeuchi, Naoko Sakuragawa, Teruyuki Ueda, Taku Sanada, Kazuo Notsumata, Takuro Terada

**Affiliations:** 1 Department of Diagnostic Radiology, Fukui-ken Saiseikai Hospital, 7-1, Funabashi, Wadanaka-cho, Fukui 918-8503, Japan; m-yamashiro@fukui.saiseikai.or.jp (M.Y.); ikeda.rie8022@fukui.saiseikai.or.jp (R.I.); matsumoto.junichi0010@fukui.saiseikai.or.jp (J.M.); takeuchi.kiyotaka1017@fukui.saiseikai.or.jp (K.T.); n-sakuragawa@fukui.saiseikai.or.jp (N.S.); 2 Department of Hepatology, Fukui-ken Saiseikai Hospital, 7-1, Funabashi, Wadanaka-cho, Fukui 918-8503, Japan; t-ueda@fukui.saiseikai.or.jp (T.U.); t-sanada@fukui.saiseikai.or.jp (T.S.); k-notsumata@fukui.saiseikai.or.jp (K.N.); 3 Department of Hepatobiliary Pancreatic Surgery, Fukui-ken Saiseikai Hospital, 7-1, Funabashi, Wadanaka-cho, Fukui 918-8503, Japan; t-terada@fukui.saiseikai.or.jp

**Keywords:** hepatocellular carcinoma, transarterial chemoembolization, tumor burden, computer application software

## Abstract

**Simple Summary:**

Although transarterial chemoebolization (TACE) is indicated for small hepatocellular carcinoma (HCC) as a second choice, TACE for small HCC is frequently difficult and less effective because of less hypervascularity and the presence of tumor portions receiving a dual blood supply. The aim of this study was to evaluate the efficacy of superselective cTACE under guidance software for patients with HCC within three lesions smaller than 3 cm. By using TACE guidance software, 81.2% of HCC lesions could be completely embolized and the cumulative local tumor progression rates in 303 tumors at 1, 3, 5, and 7 years were 17.8, 27.8, 32.0, and 36.0%, respectively. The 1-, 3-, 5-, and 7-year overall and recurrence-free survival rates in 175 patients were 97.1 and 68.7, 82.8 and 34.9, 64.8 and 20.2, and 45.3 and 17.3%, respectively. Our results indicate the efficacy of superselective cTACE using guidance software for HCC within three lesions smaller than 3 cm.

**Abstract:**

The indication of transarterial chemoembolization (TACE) has advanced to hepatocellular carcinoma (HCC) of Barcelona Clinic Liver Cancer (BCLC) stage A when surgical resection (SR), thermal ablation, and bridging to transplantation are contraindicated; however, TACE for small HCC is frequently difficult and ineffective because of less hypervascularity and the presence of tumor portions receiving a dual blood supply. Here, we report outcomes of superselective conventional TACE (cTACE) for 259 patients with HCCs within three lesions smaller than 3 cm using guidance software. Automated tumor feeder detection (AFD) functionality was applied to identify tumor feeders on cone-beam computed tomography during hepatic arteriography (CBCTHA) data. When it failed, the feeder was identified by manual feeder detection functionality and/or selective angiography and CBCTHA. Regarding the technical success in 382 tumors (mean diameter, 17.2 ± 5.9 mm), 310 (81.2%) were completely embolized with a safety margin (5 mm wide for HCC ≤25 mm and 10 mm wide for HCC >25 mm). In 61 (16.0%), the entire tumor was embolized but the safety margin was not uniformly obtained. The entire tumor was not embolized in 11 (2.9%). Regarding the tumor response at 2–3 months after cTACE in 303 tumors excluding those treated with combined radiofrequency ablation (RFA) or SR and lost to follow-up, 287 (94.7%) were classified into complete response, seven (2.3%) into partial response, and nine (3.0%) into stable disease. The mean follow-up period was 44.9 ± 27.6 months (range, 1–109) and the cumulative local tumor progression rates at 1, 3, 5, and 7 years were 17.8, 27.8, 32.0, and 36.0%, respectively. The 1-, 3-, 5-, and 7-year overall and recurrence-free survival rates in 175 patients, excluding those with Child–Pugh C class, who died of other malignancies, or who underwent combined RFA or hepatic resection, were 97.1 and 68.7, 82.8 and 34.9, 64.8 and 20.2, and 45.3 and 17.3%, respectively. Our results indicate the efficacy of superselective cTACE using guidance software for HCC within three lesions smaller than 3 cm.

## 1. Introduction

Transarterial chemoembolization (TACE) is widely performed for the treatment of unresectable hepatocellular carcinoma (HCC). There are two major techniques of TACE: conventional TACE (cTACE) using iodized oil (Lipiodol 480, Guerbet Japan, Tokyo, Japan) and gelatin sponge (GS) particles; and TACE using drug-eluting beads (DEB-TACE). Although several randomized controlled trials (RCT) conducted in Europe have indicated no significant differences in therapeutic effects between the two techniques [1,2,3], DEB-TACE is widely accepted in Western countries because it can provide an amelioration of quality of life due to its lower liver toxicity, although its routine use is slightly more expensive over the entire post-TACE life span [4]. On the other hand, cTACE is mainly performed in Asian countries. Its costs are lower [4]; however, cTACE damages the normal liver more severely compared with DEB-TACE [1,3,4,5], especially when it is performed non-selectively. The latest RCT conducted in Japan showed the superiority of selective cTACE over selective DEB-TACE for local tumor control [5]. Superselective cTACE, especially ultraselective cTACE defined as cTACE at the most distal portion of the subsubsegmental hepatic artery, can necrotize not only hypervascular tumor portions but also hypovascular tumor portions and the surrounding liver, like radiofrequency ablation (RFA) [6,7,8,9], and we named this effect transarterial ablation (TAA) [10]. Therefore, cTACE should be performed as selectively as possible to increase the therapeutic effects and reduce the adverse effects.

According to the latest European Society for Medical Oncology (ESMO) and American Association for the Study of Liver Diseases (AASLD) guidelines, the indication of TACE has been expanded to HCC of Barcelona Clinic Liver Cancer (BCLC) stage 0-A (BCLC-0 and A) when surgical resection (SR), thermal ablation, and bridging to transplantation are contraindicated [11,12]. However, small HCCs are usually less hypervascular and the identification of tumor feeders, as well as tumor staining, is frequently difficult on digital subtraction angiography (DSA) [13,14]. The presence of tumor portions receiving a dual blood supply may also reduce the therapeutic effect of TACE [15]. Therefore, the efficacy of TACE for HCC of BCLC-0 and A is still uncertain.

Recently, cone-beam computed tomography (CT) (CBCT) and TACE guidance software, including automated tumor feeder detection (AFD) functionality, have been developed and have contributed to improving the technical success rates and therapeutic effects of TACE [13,16,17,18,19,20,21,22,23,24]. We hypothesized that the combination of superselective cTACE and TACE guidance software can achieve sufficient therapeutic effects on small HCC. The purpose of this study was to evaluate the efficacy of superselective cTACE under guidance software for patients with HCC within three lesions smaller than 3 cm.

## 2. Materials and Methods

Our institutional review board (IRB) approved this retrospective study and individual patient consent was waived (ID: 2021-15). Written informed consent related to TACE treatment was also obtained from each patient before the procedure.

### 2.1. Patient Selection

TACE guidance software (EmboGuide, Philips Healthcare, Best, The Netherlands) has been routinely used since September 2012, and prototype TACE guidance software (EmboGuide App, Philips Healthcare) has also been used since May 2018 (The use of prototype TACE guidance software has also been approved by our IRB [ID: 2017-091]. Between September 2012 and December 2020, superselective cTACE, mostly ultraselective cTACE, was performed on 259 consecutive patients with HCC within three lesions smaller than 3 cm who were not suitable for SR because of multiple foci/tumor location or who refused SR. One-hundred sixty-nine patient had naïve HCC and 90 had newly developed tumors after curative treatment (Cohort 1). The diagnosis of HCC was established based on imaging findings: nodular staining and washout on dynamic CT and/or gadoxetate disodium (EOB-Primovist; Bayer Healthcare, Osaka, Japan)-enhanced magnetic resonance imaging (MRI). Furthermore, nodular staining on DSA and/or CBCT during hepatic arteriography (CBCTHA), and nodular perfusion defects on CBCT during arterial portography (CBCTAP), in addition to corona enhancement on the second-phase CBCTHA, could confirm the diagnosis during TACE. Tumors that demonstrated no obvious early enhancement on arterial-phase and hypointense signal on the hepatobiliary-phase of gadoxetate disodium-enhanced MRI images were also diagnosed as hypovascular (early) HCCs [25]. The serum levels of tumor markers (α-fetoprotein [AFP] and protein induced by absence of vitamin K or antagonist-II [PIVKA-II]) were also referenced.

Tumor hypervascularity, feeder detection by AFD, grades of portal vein visualization with iodized oil, technical success of TACE, and complications were evaluated in Cohort 1. Local tumor response and local tumor progression (LTP) were evaluated in 303 tumors in 195 patients (Cohort 2) because these could not be evaluated in 65 tumors in 55 patients that were treated with combined RFA after TACE, one tumor in one patient that was resected 2 months after TACE, and 13 tumors in eight patients that were lost to follow-up without evaluation of the tumor response. Additionally, intrahepatic distant recurrence (IDR), extrahepatic lesions, and prognosis were evaluated in 175 patients (Cohort 3) after exclusion of the following patients from Cohort 2: eight patients with Child–Pugh class C liver function, eight patients who died of other malignancies (colonic carcinoma [*n* = 3], renal cell carcinoma [*n* = 1], cholangiocellular carcinoma [*n* = 1], nasopharyngeal carcinoma [*n* = 1], leukemia [*n* = 1], and malignant lymphoma [*n* = 1]), and five patients in whom at least one tumor was treated with combined RFA after initial TACE.

**Figure 1:**
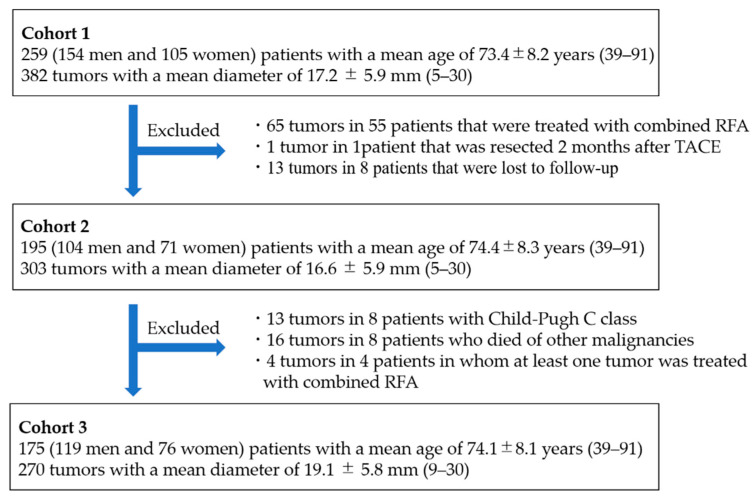
Flow chart of the study cohort. Abbreviation: RFA, radiofrequency ablation.

Patient characteristics of Cohort 1 are summarized in Table 1 and a flow chart of the study cohort is shown in Figure 1.

### 2.2. Protocol of CBCT and Angiography

All CBCT images were obtained with a 38 × 30 cm flat panel detector C-arm angiographic unit (Allura Xper FD20 or AlluraClarity Xper FD20; Philips Healthcare). Three hundred and twelve projections at 120 kV and 50–325 mA were acquired over an angular range of 240 degrees during a 5.2-s rotation of the C-arm around the patient in the expiratory phase. The 3-dimensional (3D) volume reconstructed images were displayed within 3 s after acquisition. The resulting CBCT had an isotropic resolution of 0.6 mm for a 250 × 250 × 194 mm field of view (FOV) (matrix size, 384 × 384 × 296; pixel binning, 4 × 4). The maximum radiation dose of a single CBCT scan measured on a CT phantom was 14 mGy [19].

After a 4-F 25 cm sheath introducer (Terumo, Tokyo, Japan) was inserted into the right femoral artery, a 4-F shepherd hook catheter (Angiomaster, Terumo) was navigated into the superior mesenteric artery (SMA), then CBCTAP was performed. During CBCTAP, 40 mL of contrast material (370 mg I iopamidol (Iopamiron 370; Bayer, Germany)) was injected at a rate of 3 mL/s through a catheter after the administration of 2.5 µg of prostaglandin E1 (Liple; Tanabe Mitsubishi, Osaka, Japan). The scan began 25 s after the beginning of the injection of contrast material. Then, arteriograms of SMA and the celiac artery were obtained by injection of 20 mL of contrast material at a rate of 5 mL/s. Thereafter, a common or proper hepatic arteriogram was achieved using a 4-F twist catheter (Hanako Medical, Saitama, Japan) by injecting 12 mL of contrast material at a rate of 3 mL/s. Subsequently, dual-phase CBCTHA was obtained by injection of 24 mL of contrast material at a rate of 2 mL/s. The first scan began 7 s after the beginning of the injection of contrast material and the second scan began 30 s after the end of the first scan. If the 4-F catheter could not be navigated into the common or proper hepatic artery, CBCTHA at the proper, left, or right hepatic artery was carried out using a microcatheter (Progreat ∑ [1.9-F tip], Terumo Clinical Supply, Kakamigahara, Japan, Asahi Veloute [1.7-F tip], Asahi Intecc, Seto, Japan, or Asahi Veloute Ultra [1.5-F tip], Asahi Intecc) by injecting 12 mL of contrast material at a rate of 1 mL/s. Dual-phase CBCT during arteriography (CBCTA) at the extrahepatic artery, such as the right inferior phrenic artery (IPA), was also performed by injecting 12 mL of contrast material at a rate of 1 mL/s if necessary.

### 2.3. Tumor-Feeder Detection Using TACE Guidance Software

**Figure 2:**
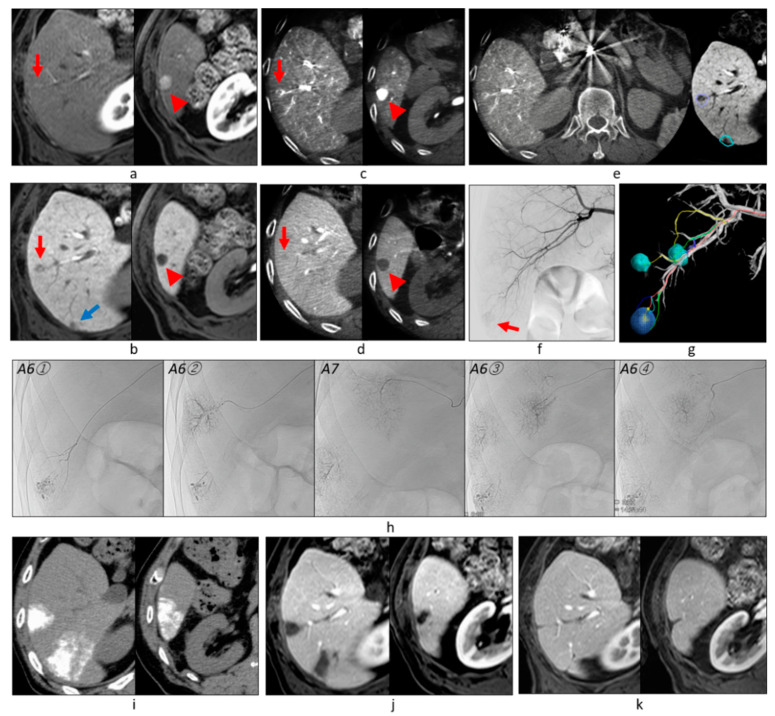
Overlay image fusion technique. (**a**) Arterial-phase images of gadoxetate disodium-enhanced MRI showed overt HCC (arrowhead) and well-differentiated HCC (red arrow). (**b**) Hepatobiliary-phase images also depicted early HCC (blue arrow). CBCTAP (**c**) and first-phase CBCTHA (**d**) showed overt HCC (arrowhead) and well-differentiated HCC (red arrow), but early HCC could not be identified. (**e**) Thus, early HCC was segmented after overlay image fusion between the first-phase CBCTHA and MRI images. (**f**) Common hepatic arteriogram showed only one tumor (arrow). (**g**) On the other hand, AFD could identify all tumor feeders. (**h**) Then, all tumor feeders were embolized without performing selective DSA of each feeder. (**i**) Iodized oil was densely accumulated in the highly limited area, including the tumors on unenhanced CT performed 1 week after TACE. (**j**) MRI performed 2 months after TACE showed that the embolized areas were necrotized regardless of tumor vascularity. (**k**) The tumors have remained well controlled for 4 years. Figure 2a–d,f–j are reprinted with permission from [24].

TACE guidance software was used at a workstation (XtraVision Interventional Workstation; Philips Healthcare) with the techniques described previously [19,21,22,23,24]. First, a virtual target lesion including a safety margin (approximately 5 mm wide for tumors <25 mm, and 10 mm wide for tumors ≥25 mm around the tumor) was created on the first-phase CBCTHA images, referencing CBCTAP and/or second-phase CBCTHA images. If the tumor could not be identified on CBCT images, it was identified by the overlay image fusion technique between the first-phase CBCTHA and conventional CT or MRI images (Figure 2). When all target lesions were created on the tumors, all potential feeders were automatically highlighted from the start position of vessel tracking to all targets on CBCTHA images and a 3D arteriogram. In EmboGuide, the start position of vessel tracking was decided using the “Set Catheter” functionality and the “Auto Detect” button was pressed to identify the tumor feeders. Then, the tumor feeders were identified within a few seconds. In EmboGuide App, the start position was automatically set on the catheter tip and the tumor feeders were highlighted instantaneously during the creation of each target lesion. When AFD could not identify tumor feeders, reanalysis was performed after enlargement of the target lesion. In EmboGuide, it was manually enlarged. In the EmboGuide App, concentric enlargement of the virtual target lesion was performed by clicking a “D+” dilation button. During this process, the highlighted tumor feeders were instantly changed according to the size of the target lesion. If excessive tumor feeders were identified, the target was again reduced in size using the “D-” button. When AFD could not identify appropriate tumor feeders, manual feeder detection (MFD) was performed using the “Add Feeder” functionality. A branch in the vicinity of the target lesion was determined as a tumor feeder and a cursor was placed on it on a 3D arteriogram or CBCTHA image. Then, the vessel was automatically traced and highlighted on the display. The process of “Add feeder” was the same for both EmboGuide and the EmboGuide App. The AFD process was usually finished within 2 min.

### 2.4. TACE Procedure

Nasal inhalation of 3 L of oxygen was performed throughout the procedure. After the acquisition of CBCTHA images, an analgesic (Pentagin, Daiichi-Sankyo, Tokyo, Japan) was administered intravenously. Then, a microcatheter was advanced into the tumor feeder distal to the subsubsegmental hepatic artery. When the branch identified by AFD was determined to be a true tumor feeder, it was embolized without performing selective DSA or CBCTHA.

**Figure 3:**
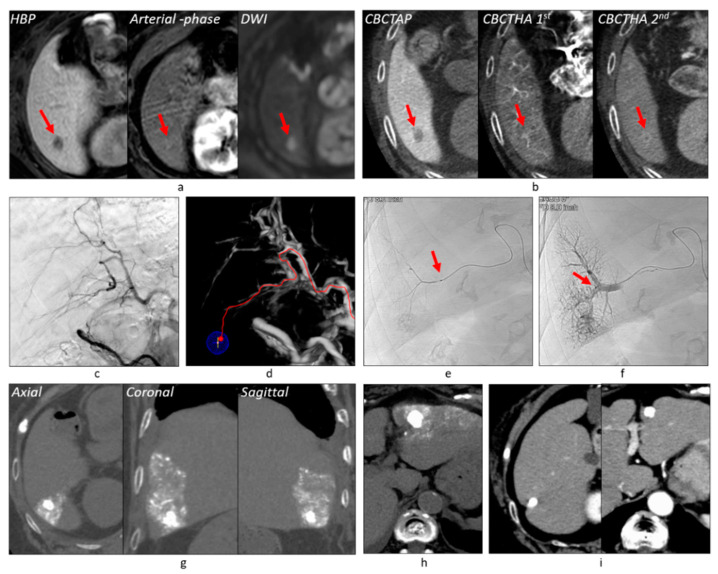
More distal advancement of a microcatheter during TACE. (**a**) Gadoxetate disodium-enhanced MRI showed a tumor of 12 mm in diameter (arrows) with a hypervascular focus. This patient also had another overt HCC 20 mm in diameter in the left hepatic lobe. (**b**) CBCT studies also depicted the tumor (arrows). Note that the second-phase CBCTHA image depicted corona enhancement around the hypervascular focus. (**c**) Common hepatic arteriogram showed no tumor stains. (**d**) AFD could identify one tumor feeder despite artifacts due to insufficient breath holding. (**e**) TACE was performed at the proximal portion of the tumor feeder (arrow), but the flow stalled during iodized oil injection. (**f**) Thus, the microcatheter was advanced more distally into the tiny tumor feeder (arrow) and iodized oil was reinjected. As a result, Grade 2 portal vein visualization could be achieved. (**g**) CT performed 1 week after TACE showed a sufficient safety margin around the tumor. (**h**) Another tumor was also successfully embolized. (**i**) Both tumors have remained well controlled for 1 year and 4 months. Abbreviations: HBP, hepatobiliary-phase; DWI, diffusion-weighted image.

After the injection of 0.5 mL of 2% lidocaine (Terumo) to prevent pain and vasospasm, a mixture of 2–8 mL of iodized oil and chemotherapeutics (10–30 mg of epirubicin (Farmorbicin; Pfizer, Tokyo, Japan) and 2–6 mg of mitomycin C (Mitomycin; Kyowa Hakko Kirin, Tokyo, Japan)) was slowly injected, followed by a GS slurry created from 1 mm-diameter GS particles (Gelpart; Nippon Kayaku, Tokyo, Japan) crushed into approximately 0.2–0.5 mm particles by pumping with a three-way stopcock valve and two 2.5 mL syringes. The ratio of chemotherapeutic solution to iodized oil was 1:3. The total amount of iodized oil was determined based on the tumor size, which was almost equal to the sum of the diameters of the target tumors. If the tumor was supplied by multiple feeders, it was separately injected according to the tumor volume supplied by each feeder. The injection of iodized oil emulsion was stopped when the flow of the tumor feeder stalled or the portal veins were clearly opacified with iodized oil near the tumor. When arterial flow was stopped before sufficient portal vein visualization, iodized oil injection was paused and 0.5 μg of prostaglandin E1 (Liple) or 0.5 mL of lidocaine was administered through the catheter to increase arterial flow and/or the microcatheter was advanced more distally and iodized oil was reinjected under a semi-wedged condition (Figure 3). We usually tried the latter technique, if possible. However, iodized oil injection was ceased when the tumor-feeding branch did not flow despite several attempts. The endpoint of GS slurry injection was complete occlusion of a tumor feeder.

### 2.5. Endpoint of the TACE Procedure

TACE was finished when all tumor stains disappeared and the tumor feeders were occluded on DSA. CBCT after TACE (LipCBCT) was not routinely performed until May 2018 when accumulation of iodized oil in the target tumor was identified on fluoroscopy. Since May 2018, LipCBCT has been routinely performed at the end of the procedure. When LipCBCT showed incomplete embolization of the tumor, TACE was added when additional tumor feeder(s) could be identified by TACE guidance software, DSA, and/or CBCT.

### 2.6. Follow-Up

All patients underwent unenhanced CT 1 week after TACE to check for iodized oil distribution in the liver and were followed up by dynamic CT and/or MRI performed every 2–3 months after TACE to investigate any tumor recurrence. In most patients, CT and MRI were performed in turn [26]. If tumor recurrence was demonstrated, additional treatment was performed according to the patient and tumor conditions, if possible.

### 2.7. Assessment

#### 2.7.1. Tumor Hypervascularity on DSA and CBCTHA

Tumor hypervascularity was evaluated based on whether tumor staining was demonstrated on a common, proper, or left or right hepatic arteriogram. If DSA showed no tumor staining, the tumor was defined as angiographically occult HCC. Additionally, it was evaluated whether the tumor had hypovascular tumor portions in the first-phase CBCTHA images.

#### 2.7.2. Detectability of Tumor-Feeders by AFD

The number of embolized arterial branches of each tumor was counted and the detectability of tumor feeders by AFD was also evaluated.

#### 2.7.3. Grades of Portal Vein Visualization with Iodized Oil

Portal vein visualization with iodized oil was evaluated by a spot digital radiograph obtained during the TACE procedure. Degrees of portal vein visualization were divided into three grades: (1) Grade 0 (no visualization), no obvious branching portal vein visualization; (2) Grade 1 (slight visualization), visualization of the portal vein adjacent to the tumor; and (3) Grade 2 (marked visualization); marked visualization of the portal veins in the whole embolized area or extending into surrounding non-embolized areas [6]. Additionally, grades of portal vein visualization were compared after categorizing them into three sizes of a microcatheter.

#### 2.7.4. Technical Success of cTACE

The embolized area was defined as the area where iodized oil was retained on unenhanced CT performed 1 week after TACE. The minimum safety margin was defined as 5 mm for tumors <25 mm and 10 mm for tumors ≥25 mm, based on a report by Sasaki et al. [27]. The embolized area was evaluated in three dimensions (axial, coronal, and sagittal views) on reviewing reconstructed CT images on the image viewer (ShadeQuest/Report, Fujifilm, Tokyo, Japan) (Figure 3). According to CT findings, the technical success of TACE was classified into three grades: (1) Grade A, the embolized area included the entire tumor with a circumferential safety margin; (2) Grade B, the embolized area included the entire tumor but the safety margin was not uniformly obtained in parts; and (3) Grade C, the embolized area did not include the entire tumor [14]. Accumulation of iodized oil in the hypovascular tumor portion was also evaluated.

#### 2.7.5. Complications

Complications were assessed based on Common Terminology Criteria for Adverse Events version 5.0 [28].

#### 2.7.6. Tumor Response, Tumor Recurrence, and Prognosis

Tumor response was evaluated on dynamic CT or MRI performed at 2–3 months after TACE using the modified Response Evaluation Criteria in Solid Tumors (mRECIST) [29]. Two radiologists (S.M. and M.Y., with 35 and 25 years of experience in liver imaging and interventional procedures, respectively) finally evaluated all CT and MRI images in consensus. Tumor recurrence was classified into three categories: LTP; IDR; and extrahepatic spread. Follow-up of this cohort was censored on 7 October 2021.

#### 2.7.7. Statistical Analysis

LTP, IDR and OS rates were calculated by the Kaplan–Meier method. The incidences of LTP between Grade A and B tumors and Grade 1–3 tumors were compared by the log-rank test. Comparisons of grades of portal vein visualization and the size of a microcatheter tip were also performed by a Chi-square test. Statistical calculations were performed using software (Excel, Microsoft, Redmond, WA, USA). All statistical analysis was two-sided and a *p*-value less than 0.05 was considered to indicate a significant difference.

## 3. Results

### 3.1. Tumor Hypervascularity on DSA and CBCTHA

**Figure 4:**
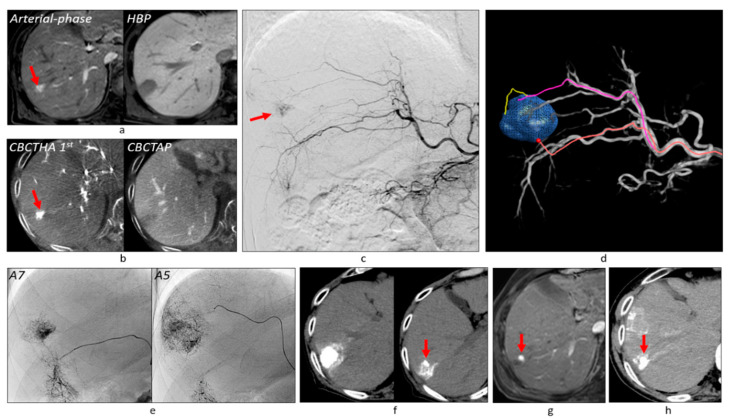
Peritumoral recurrence at the tumor portion without a safety margin. (**a**) Gadoxetate disodium-enhanced MRI showed a tumor of 27 mm in diameter with a hypervascular focus (arrow). (**b**) CBCT studies also showed the hypervascular focus (arrow) in the isovascular tumor with decreased portal blood. (**c**) Common hepatic arteriogram showed tumor stain corresponding to the hypervascular focus (arrow). (**d**) AFD could identify two tumor feeders. (**e**) Then, each tumor feeder was subsequently embolized and iodized oil was densely accumulated in the tumor and Grade 1 portal vein visualization was obtained. (**f**) However, CT performed 1 week after TACE showed that the sufficient safety margin was not obtained at the ventral side of the lower tumor portion (arrow). (**g**) Gadoxetate disodium-enhanced MRI performed 5 years and 8 months after TACE showed the recurrent tumor adjacent to the tumor portion without a safety margin (arrow). (**h**) Additional TACE was performed 6 years and 4 months after the first TACE and iodized oil was densely accumulated in the recurrent tumor (arrow) on CT performed 1 week after additional TACE. Abbreviation: HBP, hepatobiliary phase.

Among 382 tumors, 121 (31.7%) showed no tumor staining on DSA, but the first-phase CBCTHA showed hypervascularity in the entire tumor (*n* = 94) or some tumor parts (*n* = 27). Additionally, the first-phase CBCTHA images depicted hypovascular tumor portions in 30 other HCCs showing tumor staining on DSA. In total, 57 (14.9%) tumors had hypovascular tumor portions on first-phase CBCTHA images (Figure 2, Figure 3 and Figure 4).

### 3.2. Embolized Branches

**Figure 5:**
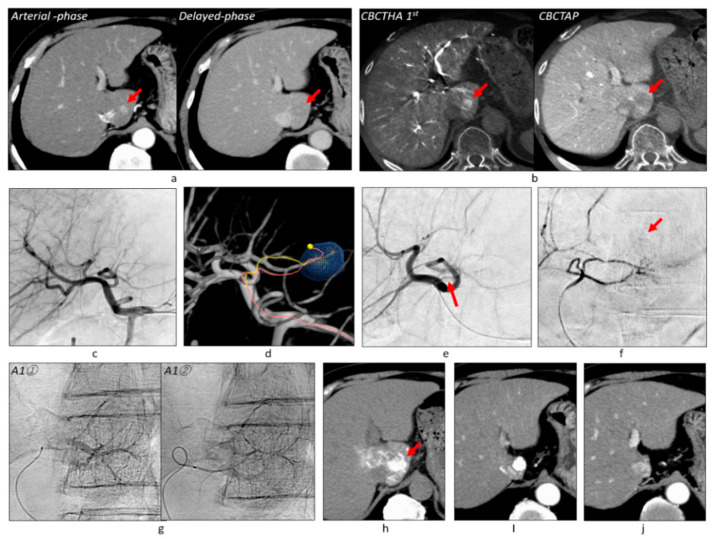
Mis-tracing of a tumor feeder. (**a**) Dynamic CT showed well-differentiated HCC of 23 mm in diameter (arrows) in the caudate lobe. The approximately 70% of tumor portions showed hypervascularity. (**b**) CBCT studies also depicted the hypovascular tumor portion with decreased portal blood around the hypervascular tumor portion (arrows). (**c**) Common hepatic arteriogram showed no tumor stains. The caudate artery (A1) was also unclear. The superior lateral subsegmental artery of the left hepatic artery arose from the left gastric artery (not shown). (**d**) AFD identified the tumor-feeding A1 arising from the medial subsegmental artery (A4). (**e**) Selective arteriogram of A4 showed A1 (arrow). (**f**) Selective arteriogram of A1 showed faint tumor staining (arrow). (**g**) Then, two branches were subsequently embolized and Grade 2 portal vein visualization could be achieved. Note that AFD mis-traced one tumor feeder (pink on Figure 5d). (**h**) Unenhanced CT performed 1 week after TACE showed dense iodized oil accumulation in almost the entire tumor (arrow). (**i**) Arterial-phase CT performed 3 months after TACE showed the disappearance of the Spiegel lobe. (**j**) The tumor has remained well controlled for 5 years and 7 months after TACE.

In total, 737 arterial branches (mean, 1.9 ± 0.9/session; range, 1–5) were embolized: 719 hepatic arterial branches and 18 extrahepatic arterial branches. Seven-hundred and 20 arterial branches (719 hepatic arterial branches and one branch of the cystic artery) were embolized as tumor feeders. Among them, 645 (89.6%) tumor feeders could be identified by AFD, although mis-tracing infrequently occurred mainly at the proximal portion (Figure 5). Seventy-five (10.4%) tumor feeders could not be identified by AFD, and they were searched for using MFD, selective DSA, and/or CBCTHA. On the other hand, 59 arterial branches, including extrahepatic arteries, such as the cystic and right gastric arteries, were misdiagnosed as tumor feeders. Additionally, 36 branches that were detected by AFD could not be identified on DSA and/or fluoroscopy during test injection of contrast material. TACE was performed proximal to the orifice of 27 uncertain tumor feeders. In the remaining nine uncertain feeders, TACE was not performed because it was considered a false feeder or embolization of a relatively large liver volume was expected by additional TACE.

**Figure 6:**
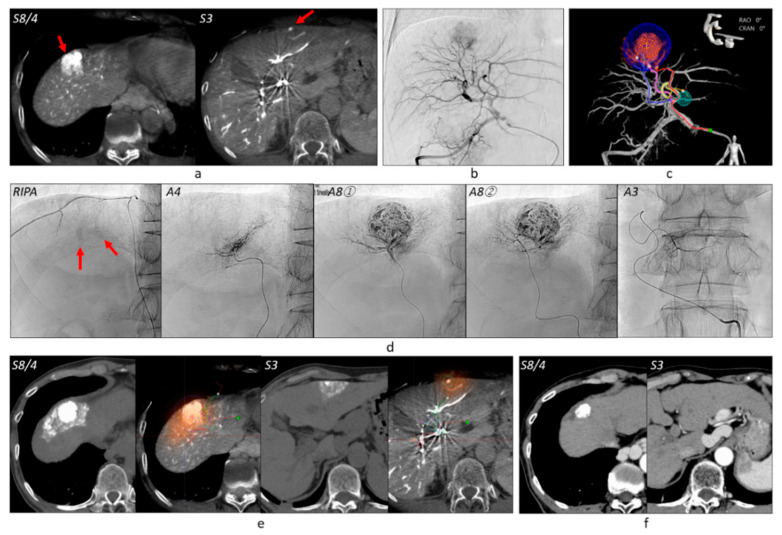
Virtual parenchymal perfusion functionality. (**a**) The first-phase CBCTHA depicted two HCCs of 28 mm and 7 mm in diameter, respectively (arrows). (**b**) The tumor in S8/4 was depicted on a common hepatic arteriogram, but a small HCC in S3\ could not be depicted. (**c**) AFD could identify three tumor feeders for the tumor in S8/4 and one feeder for the tumor in S3. (**d**) First, the anterior branch of the right IPA was embolized because the tumor was protruding into the diaphragm from the liver surface. During TACE of this branch, iodized oil accumulation in the tumor was confirmed on a spot radiograph (arrows). Then, one branch of A4 and two branches of A8 were subsequently embolized and Grade 2 portal vein visualization was obtained. Finally, one branch of A3 was embolized, but the portal veins were not opacified (Grade 0). Selective DSA of each vessel was not performed. (**e**) The virtual embolized areas and the real embolized areas on CT performed 1 week after TACE were well correlated. (**f**) The tumor in S8/4 has remained well controlled and the tumor in S3 has disappeared 4 years and 1 month after TACE. Abbreviations: S8/4, boundary between segments 8 and 4; S3, segment 3; RIPA, right inferior phrenic artery; A4, medial subsegmental artery of the left hepatic artery; A8, anterior superior subsegmental artery of the right hepatic artery; A3, superior lateral subsegmental artery of the left hepatic artery.

Blood supply to the tumor from the right superior adrenal artery that was identified by AFD using the data of CBCTA at the right IPA and iodized oil accumulation was confirmed during TACE of this vessel. Sixteen branches of the right IPA were embolized without CBCTA and AFD analysis in tumors protruding into the diaphragm or bare area from the liver surface. The branch of the right IPA was embolized first before TACE of the hepatic arterial branches (Figure 6).

### 3.3. Grades of Portal Vein Visualization

**Figure 7:**
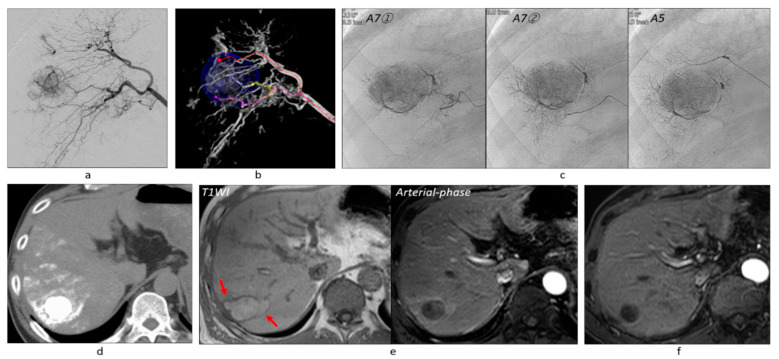
Massive peritumoal necrosis by ultraselective cTACE. (**a**) Right hepatic arteriogram showed a tumor of 30 mm in diameter. (**b**) AFD identified three tumor feeders arising from A7 and A5. (**c**) The tumor feeders were subsequently embolized and Grade 2 portal vein visualization could be achieved. (**d**) Unenhanced CT performed 1 week after TACE showed dense iodized oil accumulation in the tumor and surrounding liver. Some iodized oil was widely distributed in the anterior segment of the right hepatic lobe via the portal vein. (**e**) On T1-weighted MRI performed 2 months after TACE, the tumor and surrounding liver showed hyperintensity, indicating coagulation necrosis (arrows). The tumor showed complete necrosis on the arterial-phase of gadoxetate disodium-enhanced MRI. (**f**) Arterial-phase of gadoxetate disodium-enhanced MRI performed 8 years and 10 months after TACE also showed complete tumor necrosis. Abbreviations: A5, anterior inferior subsegmental artery of the right hepatic artery; A7, posterior superior subsegmental artery of the right hepatic artery; TWI, T1-weighted image.

Two hundred and thirty-five (61.5%) tumors were classified into Grade 2 (Figure 2, Figure 3, Figure 6 and Figure 7), 104 (27.2%) into Grade 1 (Figure 4), and 43 (11.3%) into Grade 0 (Figure 6). Grade 2 portal vein visualization was achieved in 15 (62.5%) of 24 tumors using a 1.9-F tip microcatheter (Figure 7), 104 (61.5%) of 169 tumors using a 1.7-F (Figure 2 and Figure 5), and 116 (61.4%) of 189 tumors using a 1.5-F (Figure 3 and Figure 6). There were no significant differences between the grades of portal vein visualization or size of the microcatheter tip (*p* = 0.994).

### 3.4. Technical Success of TACE

Three hundred and ten (81.2%) tumors were classified into Grade A (Figure 2, Figure 3 and Figure 5, Figure 6 and Figure 7), 61 (16.0%) into Grade B (Figure 4), and 11 (2.9%) into Grade C. Additional TACE was performed for eight of 11 Grade C tumors 4–34 months (10.8 ± 10.9) after the first TACE. At the second TACE, one residual tumor was fed by the right middle adrenal artery arising from the aorta. Four tumors were supplied by another hepatic branch. However, the remaining three were supplied by the same feeder; therefore, technical failure, such as air embolism or excessive advancement of a microcatheter, was suspected.

Regarding technical success in 121 angiographically occult HCCs, 98 (81.0%) tumors were classified into Grade A (Figure 2, Figure 3, Figure 5 and Figure 6), 18 (14.9%) into Grade B, and five (4.1%) into Grade C. Among 57 tumors with hypovascular tumor portions, 32 (56.1%) tumors showed dense accumulation in the entire or most (≥80%) hypovascular tumor portions (Figure 2, Figure 3, Figure 4 and Figure 5), 16 (28.1%) showed dense accumulation in part (<80%) of the hypovascular tumor portions, and 10 (17.5%) showed sparse or no accumulation in the hypovascular tumor portions.

### 3.5. Complications

Besides mild post-embolization syndrome and slight transit elevation of serum liver enzymes, pleural effusion and/or ascites developed or increased temporarily in 37 (14.3%) patients. In five (1.9%) patients, including three who underwent TACE of the right IPA, a partial atelectasis of the right basal lung was present. Biloma developed in two (0.8%) patients and liver abscess in one (0.4%). The patient who presented with liver abscess had a history of pancreaticoduodenectomy for pancreatic carcinoma. One biloma and liver abscess were successfully treated with percutaneous transhepatic drainage. These two (0.8%) complications were classified into Grade 4 adverse events (AE). Other complications were classified into Grade 1–2 AE.

### 3.6. Tumor Response and Local Tumor Progression

**Figure 8:**
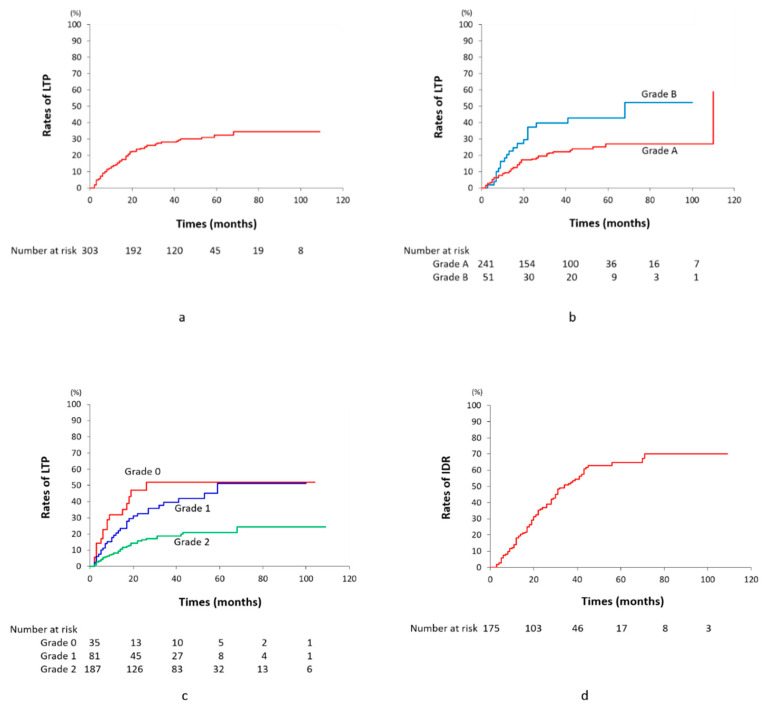
Graphs of incidences of local tumor progression in 303 tumors (**a**), between Grade A and B tumors (**b**), among Grade 0–2 tumors (**c**), and incidences of intrahepatic distant recurrence in 175 patients (**d**). Abbreviations: LTP, local tumor progression; IDR, intrahepatic distant recurrence.

Regarding the early response tumor by tumor, 287 (94.7%) of 303 tumors were classified into complete response (CR), seven (2.3%) into partial response, and nine (3.0%) into stable disease. During the follow-up period, LTP developed in 81 tumors (26.7%) with a mean interval of 14.9 ± 26.1 months (range, 2–68). Durable local CR was maintained in 222 (73.3%) tumors with a mean interval of 41.7 ± 25.8 months (range, 1–109) (Figure 2, Figure 3 and Figure 5, Figure 6 and Figure 7). The cumulative LTP rates at 1, 3, 5, and 7 years were 14.2, 27.8, 32.0, and 36.0%, respectively. The LTP rates of Grade 2, 1, and 0 tumors at 1, 3, 5, and 7 years were 8.2, 20.6, and 31.8; 18.8, 42.5, and 51.9; 20.9, 53.5, and 51.9; and 24.4, 53.5, and 51.9%, respectively. There were significant differences in LTP between Grades 2 and 1 (*p* = 0.0004) and between Grades 2 and 0 (*p* < 0.0001). There was no significant difference in LTP between Grades 1 and 0 (*p* = 0.259) (Figure 8). Regarding the relationship between the grades of portal vein visualization and LTP, 34 (18.2%) of 187 Grade 2 tumors, 31 (38.3%) of 81 Grade 1 tumors, and 16 (45.7%) of 35 Grade 0 tumors locally progressed. The LTP rates of Grade A and B tumors at 1, 3, 5, and 7 years were 9.4 and 20.5, 22.2 and 39.8, 27.0 and 42.8, and 27.0 and 51.0%, respectively. The LTP rates of Grade A tumors were significantly lower than Grade B tumors (*p* = 0.006). Regarding the relationship between technical success and LTP, 50 (20.7%) of 241 Grade A tumors, 20 (39.2%) of 51 Grade B tumors, and all 11 (100%) Grade C tumors locally progressed. Of 151 tumors with Grade 2 portal vein visualization and Grade A technical success, durable CR during the follow-up period could be achieved in 132 (87.4%) tumors with a mean interval of 42.3 ± 25.8 months (range, 1–109). Regarding the relationship between the presence of hypovascular tumor portions and LTP, 16 (28.1%) of 57 tumors locally progressed.

### 3.7. Prognosis

All patients were followed up for 3–109 months (mean, 47.6 ± 26.7). Apart from LTP, IDR developed in 92 (52.6%) of 175 patients during a mean follow-up of 21.6 ± 23.6 months (range, 3–71). The cumulative IDR rates at 1-, 3-, 5- and 7-year were 18.1, 51.7, 64.7, and 70.1%, respectively (Figure 8). Extrahepatic spread also developed in four patients (lymph node (*n* = 1), lymph node and lung (*n* = 1), bone (*n* = 1), and bone and left adrenal gland (*n* = 1)). These lesions were treated with additional TACE (*n* = 79, 1–11 times, mean; 2.5 ± 1.8), TACE plus RFA (*n* = 3, 1–2 times; mean, 1.7), TACE plus systemic therapy (*n* = 5, 1–5 TACE sessions; mean, 3 times), TACE plus systemic therapy and radiotherapy (RT) (*n* = 1, 5 TACE sessions), TACE plus RT (*n* = 3, 2–8 TACE sessions; mean, 4.3 times), TACE plus hepatic arterial infusion chemotherapy (*n* = 1, 5 TACE sessions), RFA alone (*n* = 1), and RT alone (*n* = 1).

**Figure 9:**
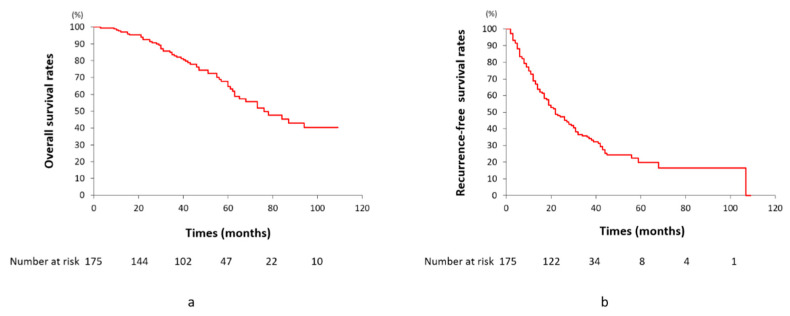
Graphs of cumulative overall survival (**a**) and recurrence-free survival rates (**b**) in 175 patients.

The cumulative OS rates at 1, 3, 5, and 7 years were 97.1, 82.8, 64.8, and 45.3%, respectively, and the median survival time (MST) was 75.4 months. Recurrence-free survival (RFS) rates at 1, 3, 5, and 7 years were 68.7, 34.9, 20.2, and 17.3%, respectively, and the MST was 22.0 months (Figure 9). Fifty-seven (32.6%) patients died between 3 and 94 months (mean, 42.5 ± 26.3) due to tumor progression (*n* = 26), hepatic failure (*n* = 13), variceal bleeding (*n* = 1), other liver-unrelated diseases (*n* = 11), and sudden death due to unknown causes (*n* = 6), including two early deaths within 1 year. Among them, 23 (40.4%) of 57 patients had no viable tumors at death. One hundred and eighteen (66.7%) patients survived from 10–109 months (mean, 50.0 ± 26.8) with (*n* = 31) or without (*n* = 87) viable tumors.

## 4. Discussion

According to the global HCC treatment algorithms, local ablation therapy, SR, or transplantation is recommended for HCC of BCLC-0 or A [11,12]. However, local ablation therapy is usually difficult for tumors adjacent to the major vessels or other organs, as well as for tumors that cannot be depicted on ultrasound or CT. SR is frequently invasive for tumors located in the central portion of the liver. Transplantation is also a limited option because of donor-liver shortages with resultant long waiting times [30]. With advancement of TACE technology, therapeutic effects of TACE have improved. As a result, TACE is now considered an alternative treatment option for small HCC [11,12].

Therapeutic effects of TACE are influenced by tumor dimensions and the number of TACE sessions. Golfieri et al. [31] reported that cTACE was most effective for naïve HCC ≤2 cm. However, small HCC may have well-differentiated tumor portions that are generally fed by both arterial and portal blood [15]. Additionally, 46% of HCCs ≤5 cm, and even 29% of HCCs ≤2.5 cm, histologically showed micrometastases around the tumor [27], mainly in the drainage area (corona) of the tumor [32,33]. They also receive a dual blood supply [15]. Therefore, non-selective cTACE has a limited therapeutic effect on HCC ≤2 cm and tumor portions receiving a dual blood supply were more likely to survive in 64% of tumors [15]. On the other hand, superselective cTACE could achieve complete tumor necrosis and peritumoral necrosis in 80–83% of tumors, including daughter nodules and capsular invasion [7,34]. Therefore, selective embolization of the tumor area including the drainage area (safety margin) is essential for small HCCs [14]. However, TACE can frequently fail to embolize the target tumors completely when it is performed using DSA alone, because identification of feeders of small HCC, as well as the tumor itself, is difficult on DSA [13,14]. In the present study, 31.7% of tumors were angiographically occult HCC. Additionally, identification of a small branch mainly supplying the safety margin is difficult even on selective DSA because tumor staining is not usually demonstrated [14]. Therefore, the use of CT or CBCT and TACE guidance software is mandatory in TACE for small HCC. AFD can identify the branches supplying not only the tumor but also safety margin by adding a sufficient safety margin to the virtual target lesion. This is a notable advantage of TACE guidance software, and AFD could identify 89.6% of tumor feeders and 81.2% of tumors could be completely embolized in the present study. Additionally, 81.0% of angiographically occult HCCs were successfully embolized and the success rate was almost equal to that of HCC showing tumor staining. This indicates that TACE guidance software can expand the indication of ultraselective cTACE to angiographically occult HCC. Another advantage of AFD is that it can reduce the procedural time and total doses of radiation exposure and contrast material [35], although it was not evaluated in the present study. However, AFD might still miss some tumor feeders, and 18.8% of tumors resulted in Grades B and C technical success. Excessive advancement of a microcatheter might also lead to incomplete embolization. To help determine the optimal catheter position, our prototype TACE guidance software has a novel function (Virtual Injection, Philips Healthcare) to visualize the virtual embolized area according to the position of the virtual catheter tip in the tumor feeder [36,37]. The preliminary results using this software were promising, and an increase in the technical success of TACE can be expected [37].

There is a relationship between LTP and grades of portal vein visualization with iodized oil. In our previous analysis, massive peritumoral necrosis was histologically observed in all tumors with Grade 2 portal vein visualization [7]. Additionally, LTP rates of the Grade 2 tumor group were significantly lower than those of Grade 1 and 0 tumor groups [6]. These results suggest that iodized oil has sufficient embolic effects to occlude the portal vein. Moreover, active injection of iodized oil into the distal hepatic artery can increase the total dose of iodized oil in the hypovascular tumor portion [8,9,10]. As a result, the therapeutic effect on small HCC can be enhanced. The present study also confirmed the same results. Additionally, the incidence of Grade 2 portal vein visualization was higher than in our previous study conducted using a 2-F tip microcatheter. Although the outer diameters of a microcatheter with a 2- and 1.9-F tip were almost equal, we suggest that advancement of microcatheter-guidewire technologies can avoid vascular spasm or injury and prevent a decrease in arterial flow via catheter manipulation. As a result, a larger amount of iodized oil can be injected into the tumor and surrounding liver. Now, we routinely use a 1.5-F tip microcatheter in TACE, although the incidence of Grade 2 portal vein visualization was almost equal among 1.5-, 1.7-, and 1.9-F tip microcatheters. A thinner microcatheter can facilitate catheterization not only into a distal portion of the hepatic and extrahepatic arteries but also into a tiny tumor feeder and can minimize the damage to the normal liver and other organs. This is a significant advantage, especially for patients with Child–Pugh class B [38]. On the other hand, the smaller microcatheter has a thinner lumen; therefore, the flow rate of contrast material is also lower. However, in our procedure, the tumor and tumor feeder are identified by CBCT data and most selective DSA can be skipped [23]; therefore, a high flow rate is not required for a microcatheter and tumor feeders can also be identified by CBCTHA data obtained using a 1.5-F microcatheter. Additionally, more distal advancement of a microcatheter can enhance the embolic effect on hypovascular HCC [8,9,10]. In the present study, iodized oil accumulation in more than 80% of the hypovascular tumor portion was achieved in 59.6% of tumors, and durable CR was also achieved in 62.5% of tumors.

There are a few reports regarding the OS and RFS of patients with HCC of BCLC-0 and A. In a report by Wang et al. [39], the 1-, 3-, and 5-year cumulative OS and RFS rates were 96.1 and 84.3, 87.8 and 59.9, and 77.2 and 50.8% in patients with HCC of BCLC-0 treated with SR, respectively, and those were 91.6 and 61.2, 73.5 and 28.3, and 57.4 and 14.1% in patients treated with RFA, respectively. The SR group had significantly better OS and RFS rates than the RFA group (*p* = 0.001 and <0.001, respectively). In patients with HCC of BCLC-A, the 1-, 3-, and 5-year cumulative OS and RFS rates were 96.1 and 84.3, 87.8 and 59.9, and 77.2 and 50.8% for the SR group and 91.6 and 61.2, 73.5 and 28.3, and 57.4 and 14.1% for the RFA group, respectively. The SR group had significantly better OS and RFS rates than the RFA group (*p* = 0.001 and <0.001, respectively). There was no significant difference in OS between RFA and SR groups (*p* = 0.088) after adjusting covariates in multivariate analysis; however, the RFA group had a significantly higher risk of recurrence than the SR group. Ryu et al. [40] reported excellent outcomes of operative microwave ablation for patients with HCC within three lesions smaller than 3 cm, and the OS and RFS rates at 1, 3, 5, and 10 years were 98 and 91, 87 and 60, 73 and 42, and 39 and 21%, respectively. Yang et al. [41] reported that the RFS rate in patients with solitary HCC ≤3 cm was significantly worse in the cTACE group than in the SR and RFA groups after inverse probability weighting, although the weighted OS was equal among the three groups. The RFS rate in the present study was better than that in a report by Yang et al. (almost equal to that of their RFA group) [41] but was also worse than that of SR and microwave ablation therapy. This might be caused by high LTP rates of TACE therapy, and it might also promote IDR [23]. As mentioned above, inflow of iodized oil into the portal vein around the tumor can reduce LTP; however, it is influenced by the vascularity of the tumor and microcirculation in the surrounding liver, because iodized oil reaches the portal vein through the drainage of the tumor and pre-existing arterioportal shunts, such as the peribiliary vascular plexus [6,9,42]. It is also influenced by the catheter position during TACE because iodized oil injection under a semi-wedged condition can increase the volume of iodized oil that flows into the portal vein [9,10]. However, the microcatheter cannot be advanced to the optimal position in some tumor feeders due to vessel angulation caused by chronic liver disease. Therefore, peritumoral necrosis cannot always be achieved, and this is a technical difficulty of TACE for small HCC.

There are several limitations to the present study. First, this was a retrospective study conducted in a single institution. Second, not all hypovascular tumors detected by gadoxetate disodium-enhanced MRI were treated. Any tumor at the periphery of the liver expected to be easily embolized might be preferentially selected. In addition, there is no consensus on the indication of TACE for hypovascular HCC. Third, the magnitude of TACE was not uniform in each tumor. When selective catheterization into the tumor feeder arising from the artery supplying a large liver volume was impossible, complete blockage of the entire embolized branches was avoided to reduce liver damage. This might markedly influence the grade of portal vein visualization and incidence of LTP. Fourth, TACE was performed for 167 patients with a single HCC lesion because most of them selected TACE treatment, not SR, and were referred to our hospital. Finally, the patient backgrounds were not very homogenous; therefore, we evaluated outcomes using an irregular approach. Technical success was evaluated in all tumors including tumors that were developed in patients with Child–Pugh C class or were treated with TACE plus RFA, because we treated all tumors with a curative intent. Additionally, we wanted to evaluate the technical success of TACE using a large number of tumors and avoid the bias that additional RFA might be performed for tumors, resulting in unsuccessful TACE. However, the patients treated with TACE plus RFA were excluded from the evaluation of LPT and prognosis, and the patients with Child–Pugh C class and patients with other malignancies that developed after TACE were also excluded from the evaluation of prognosis, because the rates of LPT and survival might be strongly influenced by such patients.

## 5. Conclusions

Superselective cTACE under guidance software has a sufficient therapeutic effect on HCC within three lesions smaller than 3 cm and can expand the indication of TACE to angiographically occult HCC and some types of hypovascular HCC.

## Figures and Tables

**Table 1:** Patient characteristics of Cohort 1.

**Patient**	**Value**
Number of patients	259
Sex, male/female	154/105
Age, y, mean (range)	73.4 ± 8.2 (39–91)
Child–Pugh Class (score) A (5/6)/B (7/8/9)/C (10)	202 (144/58)/49 (28/14/7)/8 (8)
ECOG performance status 0/1	241/18
**Etiology**	**Value**
Hepatitis C	147
Hepatitis B	51
Hepatitis B + C	8
Alcohol	14
Hepatitis C + alcohol	2
Hepatitis B + alcohol	2
Nonalcoholic steatohepatitis	10
Primary biliary cirrhosis	3
Unknown	22
**Tumor**	**Value**
Single/2/3	167/61/31
Size, mm, mean (range)	17.2 ± 5.9 mm (5–30)
AFP level, ng/mL, mean (range)	85.7 ± 396.6 (2–3950)
PIVKA-II level, mAU/mL, mean (range)	186.7 ± 1278.0 (7–18,371)
**Prior Treatment**	**Value**
None	173
Hepatectomy	16
TACE	31
RFA	13
Hepatectomy + TACE/RFA/HAIC/radiation	9/2/1/1
TACE + RFA	13
HAIC	1
HAIC + TACE	1
Radiation	2

Abbreviations: AFP, alpha fetoprotein; ECOG, European Cooperative Oncology Group; HAIC, hepatic arterial infusion chemotherapy; PIVKA-II, protein induced by absence of vitamin K or antagonist-II; RFA, radiofrequency ablation; TACE, transarterial chemoembolization.

## Data Availability

Data are available in a publicly accessible repository.
